# Urostomy-associated stomal variceal hemorrhage secondary to portal hypertension: A case report and interventional radiology–guided management

**DOI:** 10.1016/j.radcr.2026.06.139

**Published:** 2026-07-18

**Authors:** Casey Sun, Hailey Mangio, Henry Bryan, Mary Arndt, David Mina, Hakob Kocharyan, Mustafa Al-Roubaie

**Affiliations:** a Department of Radiology, University of South Florida Morsani College of Medicine, Tampa, FL, USA; b Department of Interventional Radiology, Moffit Cancer Center, Tampa, FL, USA

**Keywords:** Stomal variceal, TIPS, Portal hypertension, Urostomy

## Abstract

Stomal varices are a rare manifestation of ectopic portosystemic collateralization in patients with portal hypertension and are most commonly reported in association with ileostomies and colostomies. Stomal variceal hemorrhages are associated with significant morbidity, high rebleeding rates, and mortality of approximately 3%-4% per bleeding episode. To date, there has been limited discussion on treatment strategies for urostomy-associated stomal varices. We report a case of recurrent, transfusion-dependent bleeding from an ileal conduit urostomy in a 58-year-old man with portal hypertension secondary to metabolic dysfunction–associated steatohepatitis (MASH) and alcohol-related cirrhosis. Despite multiple interventional radiology–guided embolization procedures, bleeding persisted until definitive portal decompression was achieved with transjugular intrahepatic portosystemic shunt (TIPS) placement. This case highlights urostomy-associated stomal varices as a less familiar manifestation of ectopic variceal bleeding and emphasizes the importance of addressing portal hypertension along with isolated variceal obliteration. TIPS provided durable hemostasis after variceal embolization failure and should be considered early in refractory stomal bleeding.

## Introduction

Stomal varices, also referred to as parastomal or peristomal varices, are an uncommon form of ectopic varices that arise when portal hypertension drives hepatofugal flow through surgically created stomal venous channels. These collaterals most frequently develop in patients with ileostomies or colostomies, where mucocutaneous junctions provide a low-resistance portosystemic outflow pathway [1,2]. Cirrhosis, particularly due to metabolic liver disease, or primary sclerosing cholangitis represents the most common underlying etiology [3].

Although rare, stomal varices are clinically significant due to their propensity for recurrent and potentially life-threatening hemorrhage, with a mortality rate of approximately 3%-4% with each bleeding episode [4]. Bleeding may present as focal hemorrhage amenable to local measures or as diffuse venous oozing reflective of elevated portal venous pressures, often necessitating portal decompressive therapy [5].

Management strategies are heterogeneous and include pharmacologic therapy, local compression, sclerotherapy, percutaneous or transhepatic embolization, surgical revision, balloon-occluded retrograde transvenous obliteration (BRTO), and transjugular intrahepatic portosystemic shunt (TIPS) creation. However, residual and recurrent ectopic varices remain common when portal hypertension is not adequately addressed [6].

To date, reported cases of stomal varices have focused on gastrointestinal ostomies with limited literature on urostomy-associated stomal variceal bleeding. We present a case of refractory urostomy bleeding secondary to portal hypertension in a patient with cirrhosis, emphasizing the diagnostic challenges and the interventional radiology–guided management culminating in long-term hemostasis with TIPS.

## Case report

A 58-year-old man presented with recurrent bleeding from a permanent ileal conduit urostomy. His medical history was notable for bladder and prostate cancer treated with cystoprostatectomy and ileal conduit urinary diversion 2 years prior to presentation. Additional comorbidities included obesity (BMI 37 kg/m²), chronic kidney disease stage 3, prior venous thromboembolism on anticoagulation, and cirrhosis secondary to metabolic dysfunction–associated steatohepatitis (MASH) with ongoing alcohol use.

The patient experienced multiple hospital admissions over an 18-month period for recurrent episodes of gross hematuria via the urostomy, resulting in symptomatic anemia requiring repeated blood transfusions. He described steady, nonpulsatile bleeding from the stoma associated with fatigue and dyspnea. Social history was significant for continued alcohol consumption of approximately 3-6 beers daily with heavier intake on weekends.

On examination, the patient was hemodynamically stable (blood pressure 127/79 mmHg, heart rate 84 bpm). The urostomy appliance contained gross blood. There was no ascites, jaundice, or hepatic encephalopathy. Laboratory evaluation demonstrated severe anemia (hemoglobin 6.8 g/dL), preserved hepatic synthetic function (albumin 3.9 g/dL, INR 1.1), and chronic kidney disease (creatinine 2.0 mg/dL). The Model for end-stage liver disease (MELD) score was 12, and Child–Pugh class was A.

Contrast-enhanced CT of the abdomen and pelvis demonstrated hepatic steatosis, mild splenomegaly, and prominent stomal venous collaterals extending from the right superficial inferior epigastric vein to the ileal conduit, consistent with stomal varices (Fig. 1). Transhepatic portal venography confirmed portal hypertension with a portosystemic pressure gradient of 12 mmHg and demonstrated stomal varices draining into superficial abdominal wall veins (Fig. 2).

**Fig. 1:**
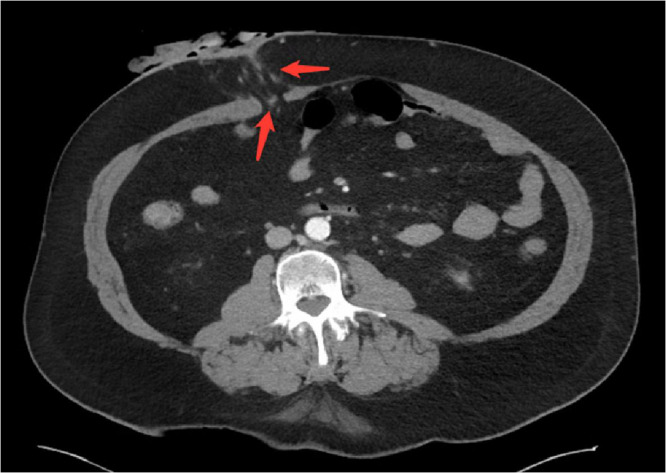
Contrast-enhanced CT abdomen demonstrates stomal venous collateralization connecting the right superficial inferior epigastric vein to the ileal conduit (red arrows).

**Fig. 2:**
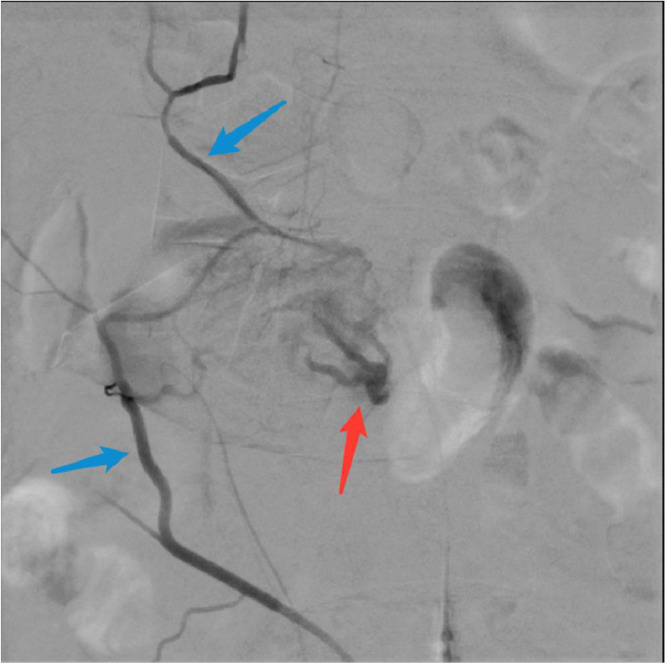
Transhepatic portal venography demonstrates stomal varices (red arrow) and drainage into superficial abdominal wall veins (blue arrows).

Endoscopic evaluation of the ileal conduit revealed mucosal congestion and vascular ectasia consistent with portal hypertensive enteropathy (PHE), as well as small grade I varices approximately 2 cm from the stoma without high-risk stigmata (Fig. 3, Fig. 4). Correlation of imaging, venography, and endoscopic findings established the diagnosis of urostomy-associated stomal variceal bleeding secondary to portal hypertension.

**Fig. 3:**
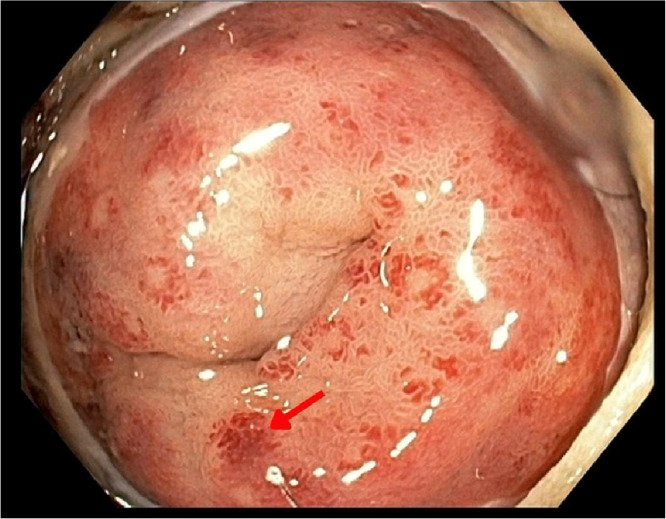
Endoscopic image of the ileal conduit demonstrates mucosal congestion and arteriovenous malformation consistent with portal hypertensive enteropathy (PHE). These findings represent sequelae of portal hypertension, with the red arrow indicating the site of recurrent bleeding.

**Fig. 4:**
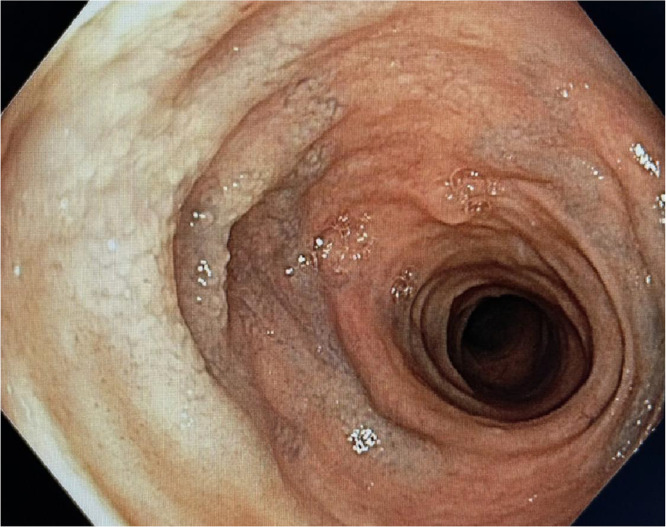
Endoscopic view of the ileal conduit approximately 2 cm from the stoma demonstrates small grade 1 varices without stigmata of recent bleeding.

### Interventional management

Initial management consisted of direct percutaneous puncture of the stomal varices under fluoroscopic guidance. Venography confirmed variceal anatomy, and embolosclerotherapy was performed using 2 cc of sodium tetradecyl sulfate (STS) mixed with 6 cc of air, followed by Gelfoam slurry. This resulted in transient hemostasis.

Bleeding recurred after 4 weeks, with active hemorrhage visualized at the urostomy stoma. A transhepatic portal venous approach was subsequently pursued, and embolization of the stomal varices was performed using 0.018-inch detachable coils and Gelfoam (Fig. 5). Despite technical success, bleeding again recurred 6 weeks later.

**Fig. 5:**
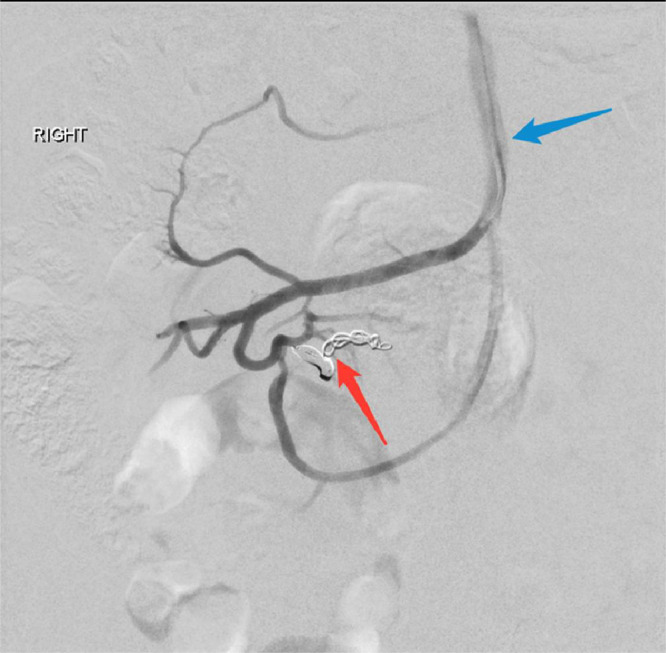
Percutaneous transhepatic portal venography via inferior mesenteric vein catheterization (blue arrow) demonstrates postcoil embolization of the stomal varices (red arrow).

Given prior evidence recommending carvedilol for treatment of increased hepatic vascular resistance, nonselective beta-blockade with carvedilol 6.25 mg twice daily was initiated for 6 weeks [7]. Despite achieving adequate heart rate reduction (66 bpm), intermittent bleeding persisted. Due to the failure of local and embolization-based therapies, portal decompression was pursued.

A TIPS was placed via a transjugular approach, reducing the portosystemic gradient from 15 mmHg to 9 mmHg (Fig. 6). Following TIPS placement, the patient developed hepatic encephalopathy 1 week postprocedure, requiring hospitalization and initiation of lactulose and rifaximin. Symptoms resolved within 48 hours, and the patient was discharged home.

**Fig. 6:**
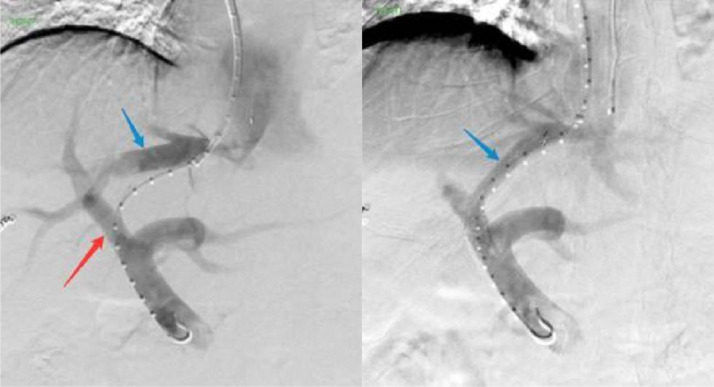
(A) Predeployment transjugular intrahepatic venography identifies the right hepatic vein (blue arrow) and right portal vein (red arrow). (B) Post-TIPS venography demonstrates the deployed stent bridging the portal and hepatic venous systems (blue arrow).

At 3-, 6-, and 12-month follow-up, Doppler ultrasound demonstrated a patent TIPS with normal flow velocities, and the patient reported no further episodes of urostomy bleeding.

## Discussion

Stomal varices are an uncommon but clinically important manifestation of ectopic portosystemic collateralization, accounting for less than 5% of all variceal bleeding episodes [8]. Their development reflects preferential decompression of elevated portal venous pressures through surgically altered venous anatomy, particularly at mucocutaneous junctions [9].

This case highlights several diagnostic challenges unique to urinary diversions. Bleeding from a urostomy may initially be attributed to urologic causes, delaying recognition of portal hypertensive hemorrhage. Multimodality evaluation, including cross-sectional imaging, portal venography, and endoscopic assessment, was essential in establishing portal hypertensive enteropathy as the underlying mechanism.

From an interventional radiology perspective, management hinges on whether therapy addresses the downstream varix or the upstream portal hypertension. Local measures and embolization techniques may provide immediate hemostasis but are associated with high rebleeding rates when portal pressures remain elevated [6,10]. In this case, both direct percutaneous embolosclerotherapy and transhepatic coil embolization failed to provide ongoing bleeding control, underscoring the limitations of isolated variceal obliteration. However, previous literature has also demonstrated that transhepatic coil embolization can be utilized for patients who experience stomal variceal hemorrhage [11]. Other alternatives such as transjugular transhepatic endovascular embolization have been demonstrated to effectively control bleeding in ileal conduit variceal bleeding, and may be considered as an alternative for patients who fail conservative management or are not immediate candidates for portal decompression [12].

TIPS provides definitive therapy by decompressing the portal venous system and reducing flow through ectopic collaterals. Prior studies have demonstrated longer bleeding-free intervals with TIPS compared to embolization alone in patients with stomal varices [13,14]. Further systemic reviews and meta-analysis of literature also demonstrate combination therapy to be more effective in stopping variceal rebleeding compared to monotherapy [15]. Despite its therapeutic advantages, TIPS was not considered earlier in the clinical course due to the patient's concerns regarding the risk of hepatic encephalopathy and the procedure's relatively invasive nature and higher risks compared with other interventions. These include hepatic encephalopathy and potential implications for transplantation, so physicians must balance these against the morbidity of recurrent bleeding and transfusion dependence [16]. For example, previous literature described a patient with recurrent parastomal variceal bleeding from a colostomy who achieved durable hemostasis following BRTO, TIPS, and percutaneous transhepatic obliteration but subsequently developed grade II hepatic encephalopathy that responded to medical management [17]. Notably, post-TIPS hepatic encephalopathy has not consistently been shown to predict decreased survival, so the risk of encephalopathy should be weighed against the substantial benefits of definitive portal decompression in patients [18]. Earlier TIPS placement may have mitigated the patient's morbidity and reduced the severity and duration of symptoms.

TIPS is primarily indicated for 2 clinical scenarios: refractory ascites, typically performed on an elective basis, and portal hypertensive bleeding, which is often managed emergently. Although a portosystemic gradient greater than 5 mmHg is considered abnormal, the target gradient following TIPS varies according to the indication. For variceal hemorrhage, the goal is generally to reduce the gradient to below 12 mmHg, a threshold associated with effective hemostasis. This aligns with previous literature demonstrating adequate hemostasis in variceal bleeding when hepatic venous pressure gradients are less than 12 mmHg or when greater than 20% of baseline [19]. Meanwhile, management of refractory ascites often requires a lower post-TIPS gradient of approximately 5-7 mmHg, although there is no standardized target for post-TIPS portal pressure gradient [20]. Some literature suggests 60%-80% reduction in portal pressure to be optimal [21]. However, excessive reduction of the portosystemic gradient may increase the risk of hepatic encephalopathy by diverting a greater proportion of portal blood flow away from hepatic detoxification pathways. In this patient, hemostasis was achieved after reduction of the portosystemic gradient to 9 mmHg, supporting prior observations that absolute gradient thresholds do not uniformly predict ectopic variceal rebleeding [22]. Although simultaneous embolization at the time of TIPS has been advocated to reduce early rebleeding [17], portal decompression with previous local varicose treatment was sufficient in this case.

This report expands the spectrum of stomal varices to include urostomies and demonstrates management principles parallel those of gastrointestinal stomal bleeding. Interventional radiologists should maintain a high index of suspicion for portal hypertensive bleeding in patients with urinary diversions and cirrhosis and consider early portal decompressive strategies in refractory cases.

## Conclusion

Urostomy-associated stomal varices represent a previously limited reported manifestation of ectopic variceal bleeding. This case demonstrates long-term hemostasis may require treatment of underlying portal hypertension in addition to isolated variceal embolization. TIPS should be considered early in patients with refractory stomal bleeding, including those with urinary diversions, when clinically appropriate.

## Patient consent

Written informed consent for publication of this case report and associated images was obtained from the patient.
